# Structural and histological changes in *Ascaridia galli* exposed *in vitro* to underutilized anthelmintic plant, *Meyna laxiflora* Robyns seed extract

**DOI:** 10.1016/j.psj.2026.107131

**Published:** 2026-05-15

**Authors:** Thoudam Bishaya Devi, N. Mohilal, Chingakham Brajakishor Singh

**Affiliations:** a Department of Zoology, Manipur University, Canchipur, Manipur, India, 795003; b Institute of Bioresource and Sustainable Development, Takyelpat, Manipur, India, 795001

**Keywords:** Anthelmintic, *Ascaridia galli*, Albendazole, *Meyna laxiflora*, Histology sections

## Abstract

The methanol seed extract of *Meyna laxiflora* Robyns was evaluated *in vitro* against the most prevalent poultry nematode, *Ascaridia galli,* to assess its anthelmintic potential employing the adult worm mortality assay. Worms were subjected to varying concentrations (0.05-100mg/mL) of the extract dissolved in physiological phosphate-buffered saline solution containing 0.1% dimethyl sulphoxide, and incubated at 37 ± 1°C. A parallel assay was performed with the broad-spectrum anthelmintic drug, Albendazole. Exposure to the extract induced complete inactivation and flaccid paralysis, culminating mortality at variable lengths of time, which revealed a dose-dependent reduction in motility and enhanced mortality. Worms treated with the plant extract at 100mg/mL, attained mortality at 7.96 ± 1.00 h, comparable to ABZ (4.51 ± 0.37 h). Cytotoxicity assays demonstrated that the extract exhibited low toxicity (<5%) against HEK293T and HeLa cells. Qualitative phytochemical screening revealed abundant phytochemicals like alkaloids, flavonoids, saponins, steroids, and terpenoids, Gas chromatography-mass spectrometry analysis identified the existence of Melezitose (51.88%), l-Glucose (7.14%), and 3-Cyclohex-3-enylpropionic acid (5.76%) as major constituents. Scanning electron microscopy of treated worms displayed extensive deformation, desquamation, and wrinkling in the cuticular topography*.* Histological examination of the worms further evidenced disintegration and disruption of the cuticle, gastro-intestinal tract, and reproductive system, thereby underscoring the potent wormicidal efficacy.

## Introduction

Gastrointestinal helminth infections are among the utmost prevalent parasitic infections affecting the large populations of both animals and humans worldwide, and are suitably documented as major veterinary and public health problems across developed and developing countries (Perry and Randolph, 1999; Hafeez, 2003). The heavy burdens of diverse poultry helminths narrow the expanding production systems for poultry byproducts derived from free-range and deep-litter systems (Permin et al., 1999; Fossum et al.*,* 2009). The roundworm *Ascaridia galli* is the most widespread helminth parasite in poultry and has increased in commercially laying hens over the past decade (Permin et al.*,* 1999; Abdelqader et al.*,* 2008; Kaufmann et al.*,* 2011b; Tarbiat et al.*,* 2016).Severely infected chickens may display clinical symptoms such as lethargy, haemorrhagic lesions, and profuse diarrhoea (Ruff, 1999; Permin et al.*,* 1998; Sharma et al.*,* 2019); while heavy infestation can cause severe intestinal blockage resulting in physical obstruction of the lumen in small intestine. Integrity of intestinal mucosa can be damaged, and nutrient utilization can be affected, which decreases weight gain (Kilpinen et al.*,* 2005; Das et al.*,* 2010), resulting in high treatment costs and a significant decrease in egg and meat production Raza et al.*,* 2016; Symeonidou et al.*,* 2018). The economic impact of *A. galli* is also largely attributed to its ability to serve as a vector for the transmission of pathogenic bacteria, notably *Salmonella enterica* and *Escherichia coli* (Chadfield et al.*,* 2001; Permin et al.*,* 2006; Abdelqader et al.*,* 2012). Chemotherapy with commercially available broad-spectrum drugs, such as albendazole, levamisole, ivermectin, benzimidazole, febendazole, etc., has high efficacy in treating *A. galli* (Yaswinski et al.*,* 2013). However, there are multiple anthelmintic resistances developed among various helminth parasites (Shalaby, 2013; Arece-García et al., 2017) and are almost cosmopolitan in distribution among domestic animals and even in some human parasites (Jabbar et al.*,* 2006), and are a major problem in poultry farming (Stear et al.*,* 2007). To mitigate drug resistance and chemical residues in poultry products, alternative strategies for anthelmintic efficacy, such as screening natural compounds, have emerged as a potential therapeutic option.

**Fig. 1:**
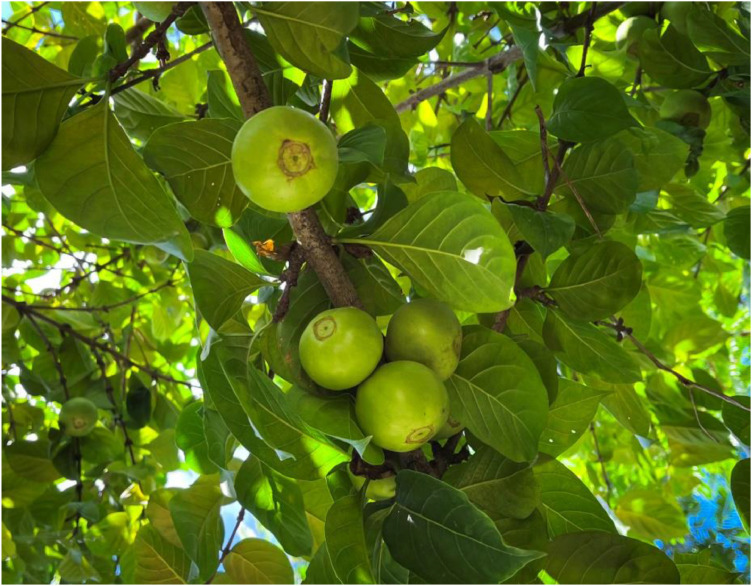
Fruits of*Meyna laxiflora* Robyns.

*Meyna laxiflora* Robyns (Fig. 1), a synonym of *Vangueria spinosa* Hook., family Rubiaceae, belongs to a group of about 6500 tree and shrub species native to the thick rainforests from Northern Bengal to Burma (Janarthanan et al.*,* 2019). It is commonly called “Muyna” in Hindi, “Muduna” in Bengali, and “Heibi” in Manipuri. It is a spiny or unarmed shrub indigenous to western Uttar Pradesh, West Bengal, Northeast India, and the Deccan Peninsula. Leaves are characterised by ovate to elliptic; lax cymose inflorescence bearing geenish-white flowers, and fruits are fleshy, subglobose, green to brown, containing 4-5 unilocular pyrenes, each enclosing a single seed (Rymbai et al.*,* 2022). Seeds are albuminous with a different testa. The fruit and leaf parts are edible, and the leaves are also used as fodder but are of inferior quality (Gupta, 2009). The fruits are of high traditional importance for treating many ailments, including inflammation and gastrointestinal disorders (Patil *et al*., 2001). Different parts are used in the treatment of boils, dysentery, diphtheria, and other conditions. In “Ayurveda”, an ancient Indian text, *M. laxiflora* is reported to possess anti-diarrhoeal and antibacterial activity (Thoreson et al.*,* 2007). The Meiteis have been using the seeds as a medicine for deworming in children and the leaves as an ingredient for ‘Singju’, an indigenous salad preparation.

So far, no scientific research/studies are available on the utilisation of this underutilised plant, *M. laxiflora* Robyns, against any parasitic worms, as well as in *A. galli.* Thereupon, to scientifically validate the *in vitro* anthelmintic activity of *M. laxiflora* seed extract relative to the common poultry nematode, *A. galli*, the present study has been taken up.

## Materials and methods

### Methanolic crude extract preparation of the seeds

The fresh fruits of *M. laxiflora* Robyns were sourced from various parts of Manipur, India, between September, 2022 to January, 2023. The plant was identified by Dr. N. Odyuo, Scientist-E & HoO at the Botanical Survey of India, Shillong, with reference number BSI/ERC/Tech/2022-23/373. The fruits were thoroughly washed with running tap water, the flesh was peeled off, the hard-coated seeds were removed, and the fruit was allowed to dry for nearly a month. Then the hard covers were crushed, the almond-like seed removed, and the covers again dried until the moisture was fully dissipated, then pulverized to a fine powder in a grinder. The powdered seeds were subjected to a cold maceration extraction technique with a 100% methanol (undiluted) solvent system for 72 hours. In a glass container, 250 g of the powdered sample was soaked in the solvent (1:10), occasionally stirring every 6 hours with a glass rod. The extract was decanted using Whatman’s filter paper No. 1, while the extracted solution was concentrated in a rotary evaporator of 40°C under reduced pressure. The MLS (*Meyna laxiflora* Robyns seed methanol extract) was refrigerated at 4°C until further use for the assay.

### Extraction yield

The percentage yield of dried seed crude extract was determined using the formula

Extraction Yield = Weight of the crude dried seed extract / Weight of the dried seed powder X 100

### Phytochemical profile

To determine the chemical constituents of the crude extract qualitatively, phytochemical profiling was performed using commonly used precipitation and coloration reactions to determine the major phytochemicals of MLS. Conventional screening tests were performed on the seed crude extract using standard procedures and reagents only to identify the constituents (Debella et al.*,* 2002; Harborne et al.*,* 2007). The screening technique was used to identify the key bioactive constituents thought to possess anthelmintic properties, *viz.*, alkaloids, flavonoids, glycosides, phenolic compounds, phlobatannins, saponins, steroids, tannins, and terpenoids. The procedures employed were chemical assays that produced colour changes when reacted with various standard reagents.

### GC-MS analysis

The Agilent 8890 standard model with a Flame Ionisation Detector (FID) was used for Gas chromatography-Mass spectrometry analysis (GC-MS) of MLS at the Sophisticated Analytical Instrument Facility (SAIF), IIT Madras, Chennai, as described below. At a constant flow rate of 1.2 mL/min, the inert gas helium (99.999%) was served as the mobile phase, and sample volume of 1 µL was employed. The capillary column was a 5% phenyl methyl siloxane HP-5 MS column with 30 m x 250 μm x 0.25 μm dimensions, and temperature spectrum of −60°C to 325°C (350°C). GC ran for a total of 53.5 minutes. The temperature of the oven rose from 75°C to 360°C. Helium gas at 1 mL/min was used to vaporise and carry the 1 µL sample down the column after injection into the port. At 70 eV, the MS was taken. The compound name, molecular weight, molecular ions (mass range), and structure of the compounds were established in the Spectral Library and Database (licensed NIST 2017 Library; software: Open Lab CDS 2.5 version), by considering their mass fragmentation patterns and retention indices (RI) within the range of 50- 600 m/z. The peak area indicated the relative percentage of each constituent.

### Cell cultures and Cytoxicity assays

The *in vitro* cytotoxicity of MLS was investigated using HeLa and HEK 293T cell lines. The cells were cultivated in Dulbecco's Modified Eagle Medium (DMEM). A total of 10^4^ cells were seeded and incubated for 24 h, followed by exposure to varying concentrations of the extract (50, 100, and 400 µg/mL) or 50 nM docetaxel, employed as a positive control, for 48 h at 37°C. Cell viability of the extract was determined by adding MTT reagent (20 mL) after 3-4 h of incubation (López-García et al., 2014; Chatterjee et al., 2022). Subsequently, 100 µL of DMSO was added, and the absorbance (A) of the samples was measured at 570 nm using a microplate reader. The percentage of cell viability was calculated as follows:

$$\text{Cell}\;\text{Viability}\;\left(\%\right)=\frac{\text{Absorbance}\;\text{of}\;\text{treated}}{\text{Absorbance}\;\text{of}\;\text{control}}\times 100$$

### *In vitro* adult worm mortality assay

*In vitro* anthelmintic effect of MLS was evaluated against adult *A. galli* (Nematoda: Ascaridiidae) obtained from freshly slaughtered naturally infected chickens, following the method of Vijaya and Yadav (2016). For *in vitro* testing, active, healthy male and female nematodes were subjected to repeated washing in 0.9% phosphate-buffered saline (PBS) at 37 ± 1°C. The test worms (n = 6) were then kept in petri dishes inside an incubator chamber at 37 ± 1°C and treated at varying concentrations (0.05, 1, 5, 10, 25, 50, and 100mg/mL of PBS) of MLS. Parallel experiment was run with same concentrations for broad-spectrum anthelmintic drug, Albendazole (ABZ). A group of worms (n = 6) maintained alone in PBS served as controls. The anthelmintic potency of the compounds was assessed by evaluating the test worms physical motility, as revealed by paralysis or mortality and was tracked every 15 minutes. For mortality confirmation of test parasites, two kinds of PBS solutions were employed: one at 37 ± 1°C and the other at 40 ± 1°C. The immobile parasites in response to soft stimulation with a soft brush, were first subjected to a warm saline solution, wherein the total loss of observable motility was interpreted as paralysis and recorded for every experimental replicate. The complete loss of perceptible motion in the parasite upon exposure to the warm solution was interpreted as a sign of paralysis, and this observation was documented for each experimental set. Similarly, when the parasites displayed no movement in this warm saline for approximately 5 minutes, they were rapidly relocated to a slightly warmer saline solution to verify their mortality. Parasite mortality was presumed to have happened when all signs of movement had stoped, even after exposure to slightly warmer saline solution for 30 minutes. The time to mortality of test parasites was documented for each experiment. The experimental protocol was performed in triplicate, employing six test worms per dose for each test agent or control group.

### Tissue preparation for histological study

Roundworms procured from the petridishes per group were dissected for histological sections from the mid-segment of the body, employing sterile instruments. The excised segments were immersed in a 10% neutral buffered formalin solution for fixation and subsequently washed with deionized water. Thereafter, the samples were subjected to dehydration through a graded ethanol series viz., 50%, 70%, 80%, 90%, and 100%, cleared in xylol, and embedded in paraffin wax. Utilizing LEICA RM2125 RTS microtome model, the worms were transversely sectioned to a thickness of 5-8 µm. Following sectioning, the tissue sections were affixed on albumi*n-*coated glass slides, deparaffinised by three successive immersions in xylene (I, II, and III), and then rehydrated through graded ethanol (100%, 90%, 80%, 70%), to permit the reintroduction of water for staining. The sections were rinsed with deionized water, stained with hematoxylin, washed with 1% acid alcohol, and again counterstained with eosin. The stained slides were then rinsed with water and mounted with a cover slip using DPX mounting medium (Lalchhandama, 2010; Darmawi et al., 2012, Darmawi et al., 2013; Isaac, 2023).Histological alterations in the tissue of *A. galli* were examined every section using light microscopy (Olympus CH 20) using a 10X, and 40X eyepiece and was photographed.

### Scanning electron microscopy (SEM)

For SEM imaging, the control and treated parasites were subjected to thorough washing in PBS followed by primary fixation and 4% glutaraldehyde buffered prepared in 0.1 M sodium cacodylate (pH 7.2) at 4°C for 4 h. Subsequently, secondary fixation was performed in 1% Osmium Tetraoxide (OsO_4_) in the same buffer for 1 h at 4°C. The specimens were then dehydrated through a graded acetone series, followed by the drying in Tetramethylsilane (TMS) following the standard method of Dey et al. (1989) remodelled by Roy and Tandon (1991) and permitted to air-dry at ambient temperature (25°C). The dried specimens were thereafter sputter-coated with gold using a JFC 1100 (JEOL) ion*-*sputter coater under a low vacuum (10^-3^ Torr). The gold-coated specimens were examined with a JEOL JSM 6360 SEM (JEOL Ltd., Tokyo, Japan) and QUANTA 250 SEM operated at an accelerating voltage of 15 kV.

### Statistical analysis

All the experimental data were represented as mean ± standard deviation (SD). Origin 2018 was used to graphically represent the data. All data were evaluated by using one-way analysis of variance (ANOVA) followed by Tukey post hoc test. Results with p < 0.001 were deemed statistically significant.

## Results

### Extraction yield and phytochemical profile of M. laxiflora Robyns

In the present study, *M. laxiflora* Robyns seeds extracted in 100% methanol showed a very low extraction yield of 4.37% (based on dry weight). The qualitative phytochemicals investigation of MLS exhibited the presence of various secondary metabolites *viz.*, alkaloids, saponins, steroids, terpenoids, with high degree of precipitation (+++) as provided in Table 1. Phenolic compounds, tannins, flavonoids, glycosides were also detected, however, phlobatannins and anthraquinones showed negativity and this might be because of their low solubility in methanol solvent.

**Table 1:** Preliminary qualitative tests for phytochemical screening of *Meyna laxiflora* Robyns methanolic seed extract (MLS).

Name of PSM	Name of the test	Crude MLS extract
Alkaloids	Dragendroff’s/ Kraut’s test	++
	Hager’s test	+++
Anthraquinones	Borntrager’s test	-
	HCl test	-
Flavonoids	Lead acetate test	++
Glycosides	Liebermann-Burchard’s test	++
Phenolics	Ferric chloride test	+
Phlobatannins	HCl test	-
Saponins	Foam test	+++
Steroids	Test for steroid	+++
Tannins	Brayner’s test	+
Terpenoids	Salkowski ‘s test	+++

Note: PSM: Plant secondary metabolites; **+++** = highest constituent; ++ = moderate constituent; + = least constituent; **—** = absent of various phytochemicals are represented. All the tests were performed in triplicate with two different extract preparations.

### GC-MS analysis

**Fig. 2:**
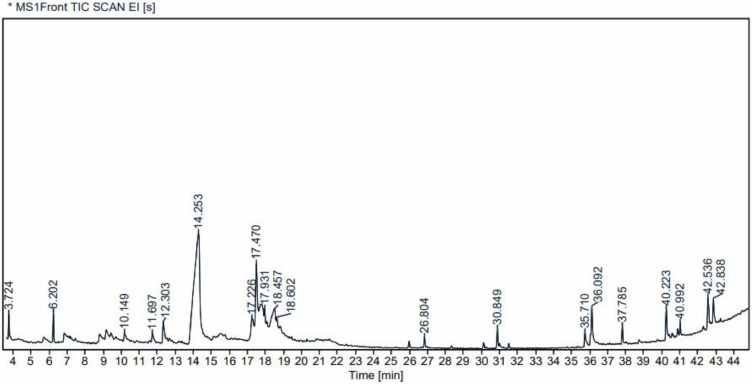
GC-MS chromatogram of *Meyna laxiflora* Robyns seed methanol extract.

Gas chromatogram of MLS had been shown in Fig. 2, and the list of chemical compounds identified through mass spectra is exhibited as provided in Table 2. Total of 19 compounds were identified, among these, Melezitose (α-d-glucopyranosyl-(1->3)-β-d-fructofuranosyl-(2->1)-α-d-glucopyranoside, a non reducing trisaccharide sugar was found to be the most abundant compound with relative abundance of 51.88%, followed by l-Glucose (7.14% & 5.51%), an enantiomer of d-Glucose; 3-Cyclohex-3-enylpropionic acid (5.76%), a volatile compound belonging to unsaturated fatty acid; and 1-(2-Acetoxyethyl)-3,6- diazahomoadamanta*n-*9- one oxime (3.53%), a bioactive nitrogen containing heterocyle compound. Furthermore, fatty acid esters *viz.*, Valeric acid, tridecyl ester (1.34%), Hexadecanoic acid, 2- hydroxy‑1-(hydroxymethyl) ethyl ester (1.31%), Octadecanoic acid, 2- hydroxyl‑1-(-(hydroxymethyl) ethyl ester (0.989%); bioactive ester *viz.*, Cyclohexanecarboxylic acid, 2- hydroxyl‑, ethyl ester (1.79%); Acetic acid, trifluoro-, octahydro-4‑hydroxy-1,5- methano-1H (2.76%), a bridged polycyclic ester under the class of terpenoiids were also detected. Two alkaloids, 1,6-Dimethyl-8- [hydroxymethyl]- tetrahydroquinoline (0.64%) and 1,2-Cinnolinedicarboxylic acid, 1,2,3,5,6,7,8,8a octahydro (2.50%) were detected. Adding on, Cyclopentanecarboxylic acid, 1-(2-butenyl)-2-oxo-, ethylester (1.93) under the class of terpenoid or sesquiterpenoid, was determined. *n-*hexadecanoic acid (0.88%), a bioactive saturated fatty acid reported to act as an anthelmintic (Lalthanpuii et al.*,* 2020; Khan et al.*,* 2024), was also determined. The ion chromatograms of the four most abundant compounds were shown in supplementary Fig. S1-S4.

**Table 2:** The main components of *Meyna laxiflora* Robyns identified in the methanolic seed extract.

Sl. No.	RT	% Peak Area	Chemical Name	Formula	MW (g/mol)
1.	3.724	1.88	Glycerin	C_3_H_8_O_3_	92.09
2.	6.202	1.89	Undecane	C_11_H_24_	156.31
3.	10.149	1.79	Cyclohexanecarboxylic acid, 2‑hydroxy-, ethyl ester	C_9_H_16_O_3_	172.22
4.	11.697	1.34	Valeric acid, tridecyl ester	C_18_H_36_O_2_	284.48
5.	12.303	3.49	Cyclohexanone, 2-ethyl-, oxime	C_8_H_15_NO	141.21
6.	14.253	51.88	Melezitose*	C_18_H_32_O_16_	504.4
7.	17.226	2.24	d-Mannose	C_6_H_12_O_6_	180.16
8.	17.470	7.14	L-Glucose	C_6_H_12_O_6_	180.16
9.	17.931	0.64	1,6-Dimethyl-8-[hydroxymethyl]−1,2,3,4- tetrahydroquinoline	C_12_H_17_NO	191.27
10.	18.457	5.51	L-Glucose	C_6_H_12_O_6_	180.16
11.	18.602	1.17	l-Gala-l-ido-octose	C_8_H_16_O_8_	240.21
12.	26.804	0.88	n-Hexadecanoic acid	C_16_H_32_O_2_	256.43
13.	30.849	1.39	Linoelaidic acid	C_18_H_32_O_2_	280.45
14.	35.710	1.93	Cyclopentanecarboxylic acid, 1-(2-butenyl)-2-oxo-, ethyl ester	C_12_H_18_O_3_	210.27
15.	36.092	5.76	3-Cyclohex-3-enylpropionic acid	C_9_H_14_O_2_	154.21
16.	37.785	1.31	Hexadecanoic acid, 2- hydroxy‑1- (hydroxymethyl)ethyl ester	C_19_H_38_O_4_	330.50
17.	40.223	2.76	Acetic acid, trifluoro-, octahydro-4‑hydroxy-1,5- methano-1H-inden-1-yl ester	C_12_H_15_F_3_O_3_	264.24
18.	40.992	0.98	Octadecanoic acid, 2- hydroxy‑1- (hydroxymethyl)ethyl ester	C_21_H_42_O_4_	358.6
19.	42.536	3.53	1-(2-Acetoxyethyl)-3,6- diazahomoadamantan-9- one oxime	C_13_H_21_N_3_O_3_	267.32
20.	42.838	2.50	1,2-Cinnolinedicarboxylic acid, 1,2,3,5,6,7,8,8aoctahydro	C_11_H_17_NO_4_	227.26

RT= Retention time, * = High abundance.

### Cytotoxicity assay

**Fig. 3:**
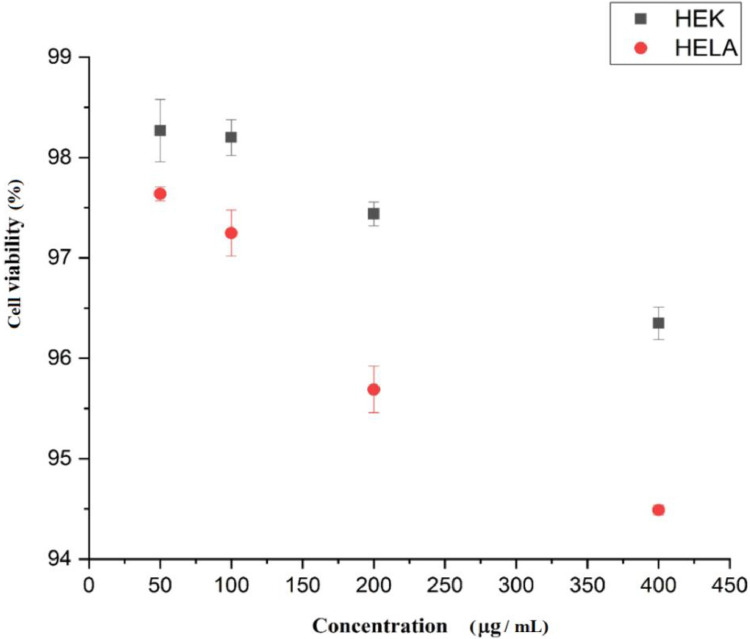
In vitro cytotoxic effect of MLS treated against HEK293T and HeLa cells. The data (mean ± SD) indicates three independent experiments.

The investigation revealed that the cell viability of HEK293T and HeLa cells at the maximum concentration of 400µg/ml was 96.35 ± 0.16% and 94.49 ± 0.05%, as shown in Fig. 3. So, MLS extract exhibited low toxicity (<5%) against these two cells even at the highest tested concentration, thus, it could be concluded that MLS extract was safe to use for anthelmintic efficacy.

### In vitro anthelmintic activity

The test model, *A. galli* incubated in MLS and ABZ, revealed a decline in the motility of the nematodes, subsequently, resulting in their death in Table 3 and supplementary Fig. S5. However, *A. galli* in the control group remained physically active, and survived robustly for about 82 h in the 0.9% PBS medium at 37±1°C following which they became immobilized and death. For both ABZ and MLS treated worms, the duration required for paralysis and death was recorded to exhibit a dose-dependent and highly significant (p ≤ 0.001) when compared to the control group. For ABZ, at the lowest dose of 0.05mg/mL, paralysis and mortality occurred at 0.60 ± 0.31 h and 0.60 ± 0.31 h, while at the highest dose of 100mg/mL, it was 0.60 ± 0.31 h and 0.60 ± 0.31 h, which show the highest anthelmintic efficacy. At a 0.05 mg/mL dose, MLS caused paralysis and mortality at 28.22 ± 0.08 h and 30.76 ± 0.12 h, while at 100mg/mL, paralysis and death occurred at 5.64 ± 0.65 h and 7.96 ± 1.00 h, respectively, which also showed high effect and was comparable with the reference drug.

**Table 3:** Dose-dependent efficacy of MLS and ABZ regarding the survival *of A. galli*.

Incubation Medium	Concentration (mg/mL)	Paralysis time (h)	Death time (h)
Control	-	-	82.67±0.94
MLS	0.05	28.22 ± 0.08	30.76 ± 0.12
	1	25.89 ± 0.43	28.5 ± 0.61
	5	19.97 ± 0.58	22.96 ± 0.7
	10	17.22 ± 0.40	19.74 ± 0.4
	25	15.56 0.45	17.86 ± 0.43
	50	11.92 ± 0.4	14.39 ± 0.38
	100	5.64 ± 0.65	7.96 ± 1.00
ABZ	0.05	39.72 ± 0.44	43.06 ± 2.1
	1	34.51 ± 0.38	38.56 ± 0.34
	5	9.57 ± 0.44	13.56 ± 0.37
	10	7.44 ± 0.35	11.37 ± 0.29
	25	5.65 ± 0.42	9.5 ± 0.37
	50	3.41 ± 0.25	7.43 ± 0.28
	100	0.60 ± 0.31	4.51 ± 0.37

Values are expressed as mean ± SD (n = 6) from 3 replicate assays. *p* value is highly significant at <0.001 for comparison of the MLS and ABZ treated parasites against the control group, Student’s *t*-test. h:hour.

### Surface topographical observations

**Fig. 4:**
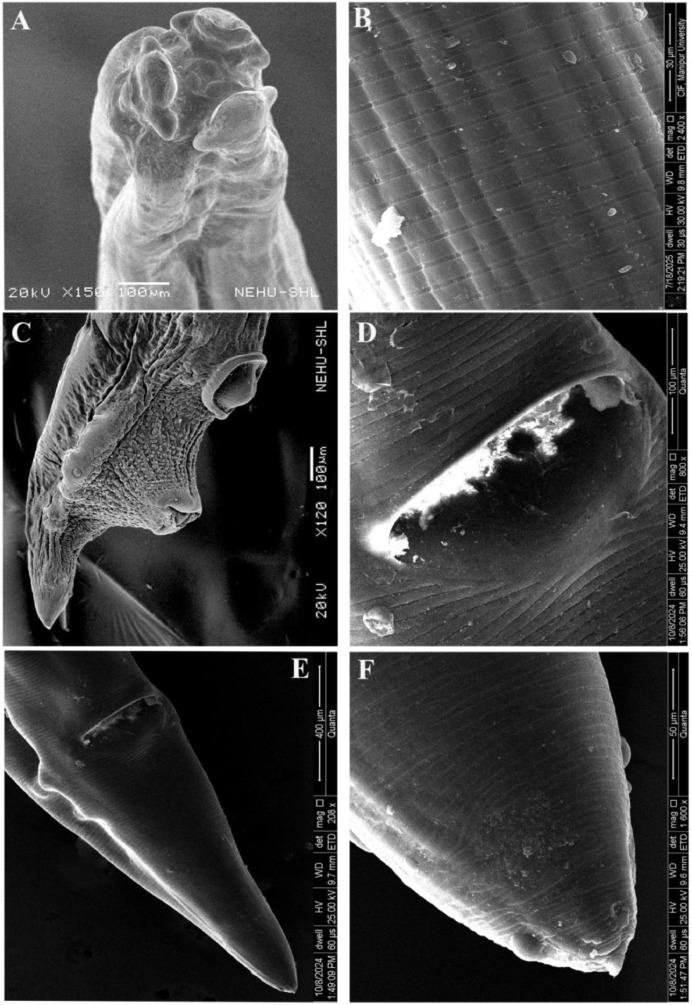
SEM images of untreated control *A. galli*. (A) Anterior end of female reviewing three denticulate lips surrounding the central mouth that leads to the digestive system. (B) Cuticular region showing annulations with distinct ridges and furrows. (C) Posterior end of male characterized by caudal alae structure, tapering tail featuring two prominent ring-like structures in the ventral portion; the anus towards the extreme end and its anterior is precloacal sucker; caudal papillae alongside anus are seen. (D) Anal opening of female with smooth surface. (E) Posterior end of female with smooth cuticle. (F) Straight and blunt tail tip.

Electron microscopic observation on control *A. galli* revealed a prominent triangular mouth surrounded by three conspicuous denticulate lips, a single dorsal and paired sub-ventral, with smooth cuticle at the extreme anterior end. Labial papillae looked more distinct in the lips (Fig. 4A). The smooth cuticular body surface was characterized by continuous transversely striated annulations running from the head region to the posterior portion. They appeared more prominent and well developed than the deep transverse grooves, namely incisures, hence, giving the body a segmented appearance (Fig. 4B). A detailed view of the cuticular surface showed more minute subdivisions of annulus into interrupted parallel concentric rings, the subannuli that ran completely around the cylindrical body through the entire length except at the very tip. The male posterior region was comparatively more complex and elaborate, having slightly inflated ill-developed caudal alae with normal cloacal protrusion. The ventral region had two distinct prominent apertures *viz.*, anus towards the extremity and preanal or precloacal sucker just anterior to it (Fig. 4C). Moreover, on the ventral side of the tail, numerous knob like structure namely, caudal papillae or phasmids were present on either side of the anus which functions as sensory organs (Fig. 4C). The posterior region of female *A. galli* featured a prominent anal opening, just prior to the extremity with a pair of papillae adjacent to its apex (Fig. 4D, E). The tail area was linear and obtusely rounded, and the cuticle was without annulations (Fig. 4F).

**Fig. 5:**
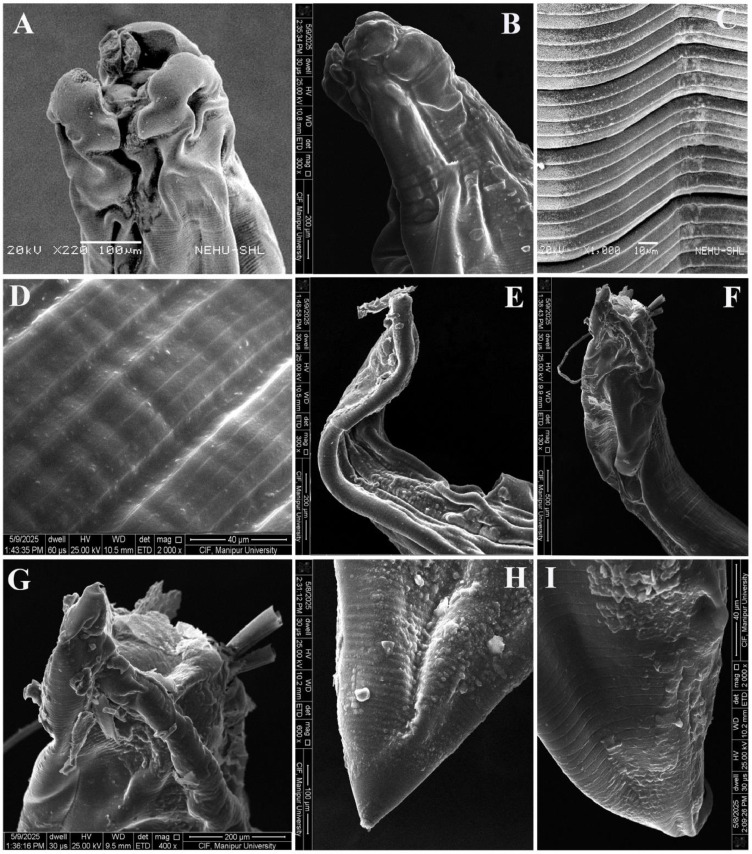
SEM images of *A. galli* treated with 100mg/mL ABZ show severe deformity, wrinkled & collapsed lips and body surface on both female anterior part (A), and male anterior part (B). (C) Cuticle in the middle portion showing lighter and darker bands with deep furrow. (D) Zoom view of cuticle; widened gap between the two annuli; wrinkled sub-annuli. (E) Female posterior part with deformed and flattened body along with wide-open anus. (F) & (G) Male posterior part with deformed caudal alae, protruded spicules, and exfoliated cuticle. Cuticular small folds with lesions were seen on the zoom view of male tail tip (H), and female tail tip (I).

The worms treated with reference drug ABZ (100mg/mL), showed significant deformation and disorganisation around the cuticular surface and mouthparts. The lips appeared collapsed, loosened, and highly folded (Fig. 5A, B); however, looked smoother and less wrinkled compared to MLS treated parasites. Both annules and subannules appeared prominently visible (Fig. 5C, D); but, the irregularities were observed less compared to MLS treated worms. The posterior part of the female looked flattened, highly folded, with a broadened anal opening, along with small bumps all over the surface on both the dorsal and ventral sides (Fig. 5E). The caudal alae were deformed and roughened, exhibiting numerous folds and evident cuticular exfoliation accompanied by protruding spicules (Fig. 5F, G). Flattened, small folds together with cuticular bumps could be seen on the tips of both female and male worms treated with ABZ, as shown in Fig. 5H, I.

**Fig. 6:**
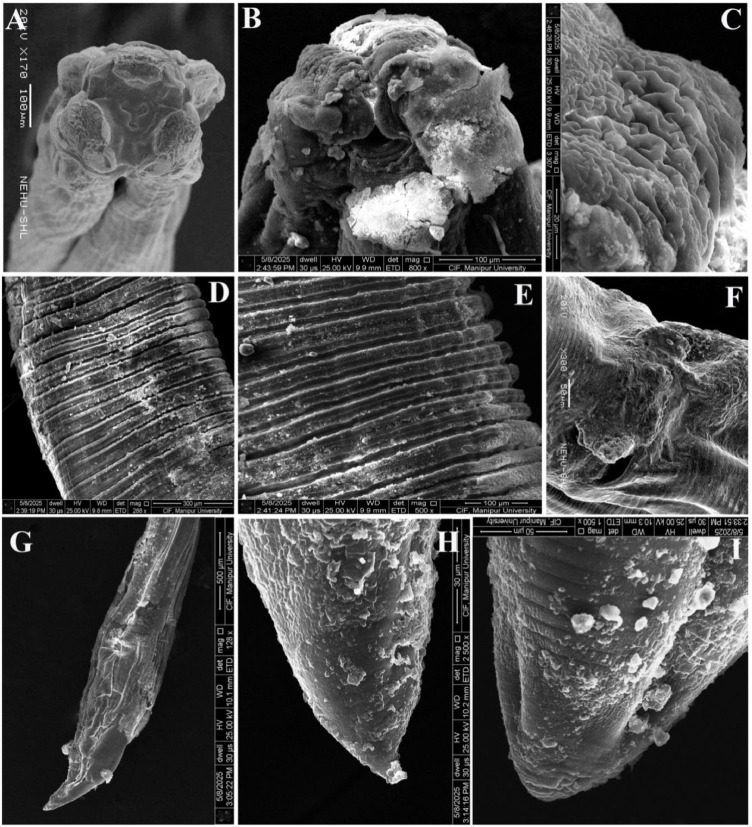
SEM images of *A. galli* treated with 100mg/mL MLS show severe deformity, wrinkled, collapsed of lips and body surface on both (A) Female anterior part; lips deformed, rough, wrinkled, expanded outward with protruberant mouth cavity; body surface wrinkled with many longitudinal folds. (B) Male anterior part; lips severely exfoliated, deformed, roughed, shrunken. (C) Male lips zoom view; severely folded rough cuticle resembling cerebriform structure. (D), (E) Cuticle in the middle portion showing desquamated epicuticle with widened, deep furrow; annulations and sub-annulations irregular. (F) Female anal opening, roughed and wrinkled. (G), (H) Male posterior end and tail tip; flattened and shrinked with eroded surface. (I) Zoom view of female tail tip showing cuticular erosion and longitudinal folds.

The *in vitro* impact of MLS on *A. galli* treated with 100 mg/mL dose showed significant deformities in the worm’s fine surface morphology. The three lips that guarded the mouth opening, along with the buccal cavity, were collapsed and loosened, showing apparent irregular wrinkles over the surface at the anterior end part of the female and male *A. galli* (Fig. 6A,B). Due to severe shrinkage, highly crumpled and exfoliated cuticule deformed like a cerebriform structure were seen on the smooth lips, labial papillae were not seen (Fig. 6B,C). The cuticle developed short longitudinal folds all over the body. The cuticle also showed significant disorganisation and aberrations in the normal striations pattern along with cuticular exfoliations along the body surface (Fig. 6D). The deep transverse grooves in between the annules were more wider along with irregular looking annules and subannules defining the deformities along the cuticular surface (Fig. 6E). The cuticle near the female anal opening appeared swollen and wrinkle (Fig. 6F). The male caudal alae at the posterior was flattened, deformed and cuticle in the posterior region appeared desquamated all over the surface (Fig. 6G). The posterior extremity of both the male and female worms showed minor folds, cuticular degradation, exfoliation and wrinkled over the surfaces (Fig. 6H, I).

### Histological observations

**Fig. 7:**
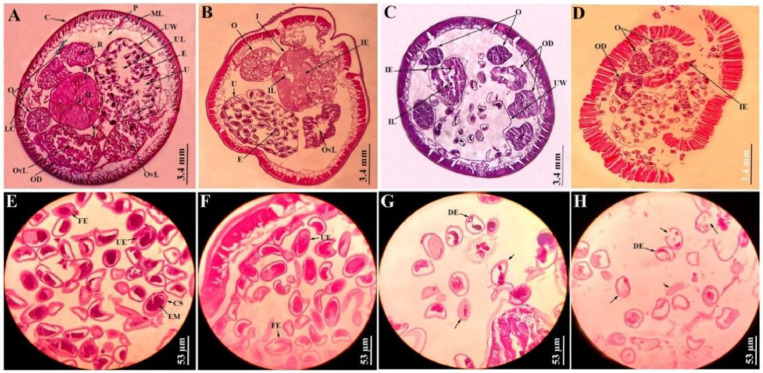
Histological sections of adult female A. galli through the body mid-part. A) Control; cuticle (C), muscle layer (ML), longitudinal excretory canal (L) surrounding the pseudocoel (P), with a triangular gut containing intestinal epithelial cells (IE) and a lumen (IL), uterus (U) surrounded by uterine wall (UW) with a lumen (UL) and numerous eggs (E), ovaries (O) with rachis (R), oviduct (OD) with a lumen (OvL) [10X]. B) 100mg/mL ABZ treated; disintegration of C and ML, shrinked intestine (I) with deformed OD [10X]. C), D) 50 & 100 mg/mL MLS treated; disintegrated and disengagement C and ML, shrinked, deformed gut and reproductive organs, disrupted and dissolved U, E scattered [10X]. E) Control uterus; fertilized (FE), unfertilized eggs (UE), encapsulated embryo (EM) by chitinous shell (CS) [40X]. F) ABZ treated uterus [40X]. G), H) 50 & 100 mg/mL MLS treated uterus; reduced eggs, numerous dead and decorticated eggs (DE), disrupting the CS, exploding the inner mass (arrow) [40X].

**Fig. 8:**
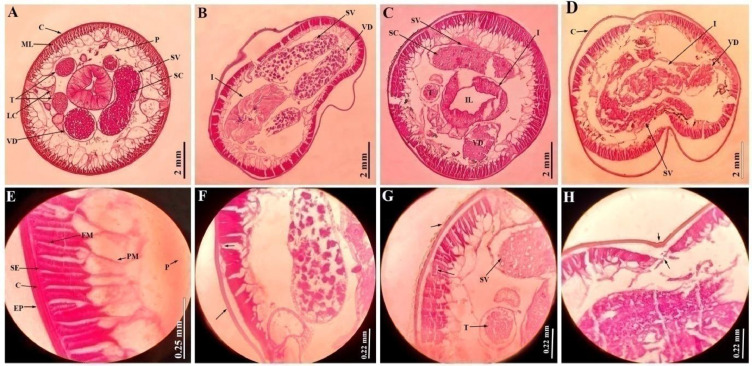
Histological sections of adult male *A. galli* through the body mid-part. A) Control; cuticle (C), muscle layer (ML), longitudinal excretory canal (L) surrounding the pseudocoel (P), with a triangular gut containing intestinal epithelial cells (IE) and a lumen (IL), seminal vesicle (SV) with spermatozoa (SC), rounded vas deferens (VD) and testes (T) [10X]. B) 100mg/mL ABZ treated; disintegration of cuticule and muscle layer, deformed intestine (I) with elongated SV and VD [10X]. C), D) 50 & 100 mg/mL MLS treated; disintegrated and disengagement C and ML, widened gut, disrupted and shrinked reproductive organs [10X]. E) Control body wall; epicuticle (EP), cuticle, syncytial epidermis (SE), fibrillar (FM) and protoplasmic layers (PM) were seen [40X]. F) 100 mg/mL ABZ treated, disintegrated, C from ML [40X]. G), H) 50 & 100 mg/mL MLS treated; disintegrated, ruptured C and ML (arrow) [40X].

The histology of *A. galli* under light microscopy reveals cylindrical body of the worm supported by the cuticle. The cuticle is a complex layer of proteinaceous rings that form a rigid, dense structure made up of several discrete concentric layers running continuously encircling the body. Just beneath the cuticle lies a syncytial epidermis, also called hypodermis, which produces an intricate layer of longitudinal muscles toward the exterior, and a connective tissue meshwork on the interior. Even the muscular layer consists of two parts: a fibrillar, tensile part running longitudinally along the epidermis, and a granular, no*n-*tensile protoplasmic part entending towards the body’s centre. The large cavity of the worm, referred to as pseudocoel, contains a large gut lying in it, which is visible in the section (Fig. 7, 8). The gut is made up of unilayer epithelial cells arranged in a circular-triangular pattern creating a visible triradiate lumen at the centre. Additionally, a sideways longitudinal excretory duct is appeared as a tiny outgrowth extending from the protoplasmic edge of the muscle into the pseudocoel. The female and male *A. galli* are bestowed with different reproductive organs (Fig. 7, 8)

Normally, in a sectional study of a female worm, the gut was encircled by a pair of ovaries, two uteri, and oviducts (Fig. 7A). However, the didelphic reproductive system, characterized by highly twisted and coiled gonads within the body cavity itself, resulted in multiple sections through the ovaries and oviducts except the uterus. In the control section of female *A. galli* (Fig. 7A), a single uterus was observed, serving as the union of the two genital tracts posterior to the vagina. The uterus wall was comparatively thicker and denser than the other reproductive parts and contained clusters of numerous egg cells, commonly known as oogonia. The egg appeared oval or somewhat polyhedral in shape and was encased in a tough chitinous protective shell, housing the yolk and embryo (Fig. 7E). Ovaries appeared solid, with the smallest diameter, non tubular with a central cellular core known as the rachis. While, oviducts appeared the same as ovaries but a little bit larger in diameter with a small lumen in the centre, the largest diameter was the uterus. Additionally, oviducts and uteri were hollow, yet they might have appeared solid owing to being packed with eggs and devoid of a rachis (Fig. 7A). *A. galli* male reproductive system was linear, comprising of a single testis that led to a vas deferens, a seminal vesicle for storage, and a narrow ejaculatory duct that opens into a cloaca.

The female worm treated with 100mg/mL ABZ, showed cuticular disintegration with significant disruptions at the underlying syncytial epidermis, along with the breakage of the associated muscle layer (Fig. 7B). The intestinal epithelial cell appeared slightly shrunkened and contracted, while the oviduct was completely deformed (Fig. 7B). Uterine wall was seen intact along with fertilized and unfertilized eggs (Fig. 7F). Fig. 7C, showed MLS treated section against female *A. galli* with 50 mg/mL dose, indicating disengagement of muscle layers from one another, and the intestinal epithelial wall was totally disintegrated from the epithelial layers, causing the cells to diffuse and contract. The ovaries and oviducts appeared deformed, shrivelled, and even the rachises were diffused. Due to the collapse of the muscular wall, the uterus was entirely disrupted, leading to the scattering of eggs all over the pseudocoel (Fig. 7C). Eggs reduced drastically in number, while many dead and decorticated eggs were seen (Fig. 7G). The female worm treated with 100mg/mL dose showed more damage than the 50mg/mL treated ones, showing total cuticular disintegration with disruption in the muscle layers; intestinal lumen contracted, intestine shrinked and got elongated; ovaries, oviducts were deformed (Fig. 7D). Uterus was deformed, ruptured, scattering numerous dead and decorticated eggs through the body cavity, even the egg shells ruptured expelling all the inner mass in the pseudocoel (Fig. 7H).

In the control section of the male *A. galli* (Fig. 8A), the gut was encircled by a prominent circular seminal vesicle together with a vas deferens, and numerous testes were visible due to their highly coiled nature. Moreover, the seminal vesicle was filled with spermatozoa, the male gametes that appear as small granular bodies. Fig. 8E, showed the body wall with clear intact of cuticular and longitudinal muscle layers. The male worm treated with 100 mg/mL ABZ, shows cuticular desquamation with significant disruptions at the underlying syncytial epidermis, along with the breakage of the associated muscle layer (Fig. 8B). The intestine, seminal vesicle and vas deferens seem elongated, ruptured, resulting in deformed no*n-*flagellated amoeboid sperm cells (Fig. 8B). The muscle fibres in the longitudinal muscle layer were disengaged from one another (Fig. 8F). The male *A. galli* sections treated with MLS 50 and 100mg/mL, show the highest histological alterations (Fig. 8C, D). In 50mg/mL MLS treated section, the intestinal lumen became wider and broader, the epithelial cells disintegrates (Fig. 8G), the seminal vesicle and vas deferens were disrupted, while the epithelial wall of the testes completely disintegrates, and was detached from the cytoplasmic rachis. Moreover, in 100 mg/mL MLS treated section, complete disintegration and disruption of the cuticle from the syncytial epidermis was seen (Fig. 8H). Muscles were disengaged along with the breakage of the associated muscle layers, the gut and reproductive systems were completely altered, deformed, shrivelled, and significant deformity were visible in the section (Fig. 8D).

## Discussion

The present research represents the investigation of anthelmintic potential inherent in an erstwhile underutilised plant, *Meyna laxiflora* Robyns, profusely found in Manipur, India, which bears a deeply entrenched cultural significance attributed to its remarkable ethnomedicinal virtues. The plant extract has been tested against the most common prevalent poultry nematode. The study reveals significant *in vitro* anthelmintic potential of MLS seed extract against the most prevalent and largest poultry nematode, *Ascaridia galli. A. galli* is the most regularly encountered issue affecting native chicken breeds, consistently causing Ascaridiasis (Shifaw et al.*,* 2021). The infected chickens show a swollened intestinal lining with hemorrhagic spots, along with lymphoid cells infiltration combined with eosinophils (Levkut et al.*,* 2022). For this gut-dwelling gastrointestinal nematode, the gut lining functions as a communication hub, leading to mucosal epithelial cell damage of the chicken’s digestive tract, thus resulting in increased mucus production, epithelial cell desquamation, mucus villi adhesion, necrosis, and goblet cell hyperplasia (Jerica et al.*,* 2024; Ritu et al.*,* 2024). *A. galli* infection disrupts nutrient uptake and also has a critical immune system modulation altering the host’s immune system response to the infection (Das et al.*,* 2021). Concurrent infections of *Ascaridia galli* and *Eimeria tenella* that were reported in *Numida meleagris*, the helmeted guinea fowl which showed severe intestinal damage and reduced nutrient absorption, leading to enteritis, poor growth, and higher infection risk. The combined impact increased economic losses, underscores the need for integrated parasite control strategies (Attia *et al*., 2025). Nevertheless, phytochemicals could be employed for prophylactic parasite management, with synthetic drugs reserved for the targeted treatment of acute infections, thereby balancing natural and potent therapies to mitigate the development of resistance (Khalil *et al*., 2025).

*A. galli* when treated with the seed methanol extract, exhibited a state of paralysis (5.64 ± 0.65 h) and eventually attained mortality (7.96 ± 1.00 h), which could be somewhat comparable to paralysis (0.60 ± 0.31 h) and death (4.51 ± 0.37 h) with ABZ at the highest tested concentration of 100mg/mL. This showed that both the MLS extract and the standard drug (ABZ) act as a dose and time-dependent manner, which was consistent with what has already been studied *in vitro* efficacies on *A. galli* using readily available ethnomedicinal plants (Mubarokah et al.*,* 2019; Nghonjuyi et al.*,* 2020; Hazarika et al.*,* 2023; Mir et al.*,* 2024a, 2024b; and Tiuria et al.*,* 2024). *Areca catechu* water extract induced structural changes leading to the death of *A. galli* (Mubarokah et al.*,* 2019). Leaf ethanol extract of *Mimosa pudica* and seed extract of *Carica papaya* were found to lower *A. galli* egg count per gram in feces and impact blood and fat levels in infected Kabir chickens by in Cameroon (Nghonjuyi et al.*,* 2020).leaf extract of the commonly known clove, *Syzygium aromaticum,* was reported to kill *A. galli* by damaging its outer layer, after exposure to 140mg/mL at 3, 6, and 9 h exposure (Anggrahini et al.*,* 2021). *Nyctanthes arbor-tristis* and *Butea monosperma* leaf extracts had potential activity against *A. galli in vivo* (Hazarika et al.*,* 2023). *Saussurea costus* crude methanolic extract inhibited worm motility in *A. galli* at 100mg/mL after 24 h of exposure (Mir et al.*,* 2024a). *Juglans regia* L. ethanol leaf extract at 100 mg/mL dose slocked the adult *A. galli* motility by 96.5% after 24 h exposure (Mir et al.*,* 2024b). Tiuria et al. (2024) reported that at 1000µg/mL, the ethanolic extract of *Phyllanthus niruri* L, *Andrographis paniculata, Curcuma xanthorrhiza* Roxb.*, Curcuma aeruginosa* Roxb reduced the motility of *A. galli. M*oreover, the presence of bioactive compounds *viz.*, Andrographolide and 5-Methoxybenzimidazole helped in damaging and sloughing off the worm’s cuticular layer. Rasheed and Aljohani (2024) reported anetholes as a major component of star anise essential oil, with the plant oil showing dose-dependent efficacy against *A. galli.*

In the present study, the MLS extract revealed the presence of numerous phytochemicals of anthelmintic potential, *viz.*, alkaloids, saponins, flavonoids, terpenoids, in higher concentrations that were known to cause damage and disruptions to the cuticular integrity, reproductive cycle, and disrupting the pH maintenance of *A. galli.* Similar to some previous studies, presence of secondary metabolites with anthelmintic activity, *viz.*, phenolics (alkaloids and tannins), terpenes (glycosides and saponins), as well as nitroge*n-*containing metabolites (alkaloids, cyanide-releasing glycosides, and no*n-*protein amino acids), highlighted the diverse potential and mechanisms of action that might be approached. The primary mechanisms of the metabolites were to damage mucopolysaccharide membrane, thus affecting the potent movement, blocking the worm’s egg production, and damaging cuticular organisation of the worm (Zaman et al.*,* 2020). Practically, for nematode control, the cuticular region was the primary focus of deworming efforts. However, actions on these complex extracellular structures depended on the actions of the metabolites thereby, showing variability in the morphological structures among the different worm genera including larvae, and adults (Tiuria et al.*,* 2024). A specific collagen and insoluble protein, cuticulin, was crucial for the cuticle’s composition and function. The cuticle comprises of three main layers: the outermost epicuticle containing insoluble proteins; the middle matrix, which is further subdivided into a fibril stratum with aromatic amino acids and a thick albumin protein layer; and the last fibrous protein layer similar to fibrin or elastin, containing carbs, lipids, and esterase catalyst (Betschart et al.*,* 2022).The extract of *M. laxiflora* Robyns had the capacity to cause disruption in these layers of the cuticle.

The GCMS analysis of MLS methanol extract demonstrated the presence of a highly abundant no*n-*reducing sweetener, Melezitose (51.88%), which had been used as a marker in entomological studies. Interestingly, *n-*Hexadecanoic acid (0.88%), which was a bioactive fatty acid that was known to exhibit significant *in vitro* anthelmintic activity, was identified from ethyl acetate tuber extract of *Corallopus epigaeus* (Ishnava and Konar, 2020). From the previous study, lots of fatty acids, viz., Tetradenoic acid, *n-* Hexadecanoic acid, Octadenoic acid, and Tetradecanoic acid, had shown deworming and oxidative stress reducing activities (Agim et al.*,* 2017; Gideon, 2015; Khan et al.*,* 2024). Octadecanoic acid, 2‑hydroxy-1-(hydroxymethyl) ethyl ester (0.98%), also known as 2-stearoylglycerol, alongside with *n-* Hexadecanoic acid, possessed anthelmintic properties that were shown in prior study (Francis et al.*,* 2021). The presence of alkaloids in MLS extract viz., 1,2-Cinnolinedicarboxylic acid, 1,2,3,5,6,7,8,8aoctahydro (2.50%) and 1,6-Dimethyl-8- [hydroxymethyl]−1,2,3,4- tetrahydroquinoline (0.64%) contributed to the significant mortality of *A. galli* compared to the control group*.* As previously mentioned Roy et al. (2010) and Badarina et al. (2017), plants with rich alkaloid content had shown high anthelmintic potential on *A. galli* by acting in the central nervous system, suppressing the sugar uptake by the worm. Additionally, the presence of two terpenoid derivatives, Acetic acid, trifluoro-, octahydro-4‑hydroxy-1,5- methano-1H and Cyclopentanecarboxylic acid (1.93%), 1-(2-butenyl)-2-oxo-, ethyl ester (1.93%) showed anthelmintic value against *A. galli* by acting on the cuticular surface, disrupting the pH balance and causing disintegration and was in conformity with previous study (Zaman et al.*,* 2020). In both male and female *A. galli,* the histological changes visualized through phytomicrography revealed evidence of MLS extract working as nematocide. In the present investigation, the SEM images revealed that the plant extract caused morphological alterations to *A. galli* throughout the cuticular surface from the mouth part till the tail tip, even the outer epicuticle surface was desquamated, which was somewhat similar to the study made by Challam et al. (2010). ABZ treated worms also showed similar deformities and disorganisation, which is consistent with similar studies Lalchhandama et al. (2009), Again, Roy et al. (2012) had similar *in vitro* efficacy of F5-2d, a compound isolated from *Acasia oxyphylla.* Images Figs. 7 and 8 showed that the present extract not only affects the outer layers but, also caused desquamation, deformation, and shrivelling of the gut and other reproductive organs, even the entire egg cell showed shrinkage and disintegration due to the breakage of the uterine wall. This has some similar results with sectional study of *A. galli* dosed with plant extracts described in few relevant works (Balqis et al., 2017a, 2017b; Lalchhandama, 2008).The present study was designed to propose an alternative control strategy for *Ascaridia galli* using the methanol extract of *M. laxiflora Robyns* as an anthelmintic plant*.* To reduce the anthelmintic resistance against broad-spectrum drugs, the use of herbal plant-based anthelmintic products might significantly vary in the poultry helminths. However, *in vivo* confirmation is much appreciated to assess the efficacy of *M. laxiflora Robyns* as an anthelmintic in the poultry birds infected with *A. galli*.

## Conclusions

The present *in vitro* experiment with methanolic extract of *Meyna laxiflora* Robyns demonstrated potent anthelmintic efficacy against adult *Ascaridia galli*, exhibiting a clear dose-dependent response. Notably, the MLS extract induced extensive morphological damage, causing deformation, disintegration, and desquamation of the worm’s entire body, including the outer cuticular region and internal tissues, as visualized via SEM and histological imaging. This structural impairment severely weakened the motility of the adult worms, leading to subsequent paralysis and death. Consequently, the findings underscored the extract’s substantial potential for the development of novel, efficacious anthelmintic therapies. For future perspective, *in vivo* validation coupled with isolation and characterization of bioactive phytochemicals from *M. laxiflora* Robyns could be essential to elucidate the precise pharmacodynamics of the plant against helminth parasites and to counteract resistance in existing anthelmintic medications.

## Ethical approval

This study was approved by the Institutional Animal Ethics Committee (Approval No. CCSEA/MU/Aca/2024/05, dated 31/12/2024) of Manipur University, Canchipur.

## CRediT authorship contribution statement

**Thoudam Bishaya Devi:** Writing – review & editing, Writing – original draft, Visualization, Validation, Software, Resources, Project administration, Methodology, Investigation, Formal analysis, Data curation. **N. Mohilal:** Visualization, Validation, Supervision, Funding acquisition, Conceptualization. **Chingakham Brajakishor Singh:** Visualization, Supervision, Funding acquisition, Formal analysis, Conceptualization.

## Disclosures

The authors declare that they have no known competing financial interests or personal relationships that could have appeared to influence the work reported in this paper.

## Data Availability

All data that support the findings of this study are included in this manuscript and are available on request from the corresponding authors.

## Appendix Supplementary materials

Image, application 1
