# Supplementary material for: Structural and histological changes in Ascaridia galli exposed in vitro to underutilized anthelmintic plant, Meyna laxiflora Robyns seed extract
Source: Poult Sci. 2026 May 15;105(9):107131. doi: 10.1016/j.psj.2026.107131 (PMC13226931; doi:10.1016/j.psj.2026.107131)

Supplemental Files

Structural and histological changes in *Ascaridia galli* exposed *in vitro* to underutilized anthelmintic plant, *Meyna laxiflora* Robyns seed extract

Thoudam Bishaya Devi[1], N. Mohilal[1] & Chingakham Brajakishor Singh[2*]

[1]Department of Zoology, Manipur University, Canchipur, Manipur, India-795003

[2]Institute of Bioresource and Sustainable Development, Takyelpat, Manipur, India -795001

Email: bishayathou@manipuruniv.ac.in, nmohilal@manipuruniv.ac.in, braja_mu@yahoo.co.in

*Corresponding author email: braja_mu@yahoo.co.in

Supplementary Figure Legends

Supplementary Figure S1. GC-MS ion chromatogram of Melezitose (51.88%) with retention time 14.25 min. Corresponding to Table 2

Supplementary Figure S2. GC-MS ion chromatogram of L-Glucose (7.14%) with retention time 17.47 min. Corresponding to Table 2

Supplementary Figure S3. GC-MS ion chromatogram of 3-Cyclohex-3-enylpropionic acid (5.76%) with retention time 36.09 min. Corresponding to Table 2

Supplementary Figure S4. GC-MS ion chromatogram of 1-(2-Acetoxyethyl)-3,6-diazahomoadamantan-9- one oxime (3.53%) with retention time 42.54 min. Corresponding to Table 2

Supplementary Figure S5. Concentration-time dependent *in vitro* anthelmintic efficacy of *Meyna laxiflora* Robyns seed methanolic extract (MLS) against *Ascaridia galli*. Corresponding to mean paralysis time and mean mortality time of Table 3.

Supplementary Figures

Supplementary Figure S1

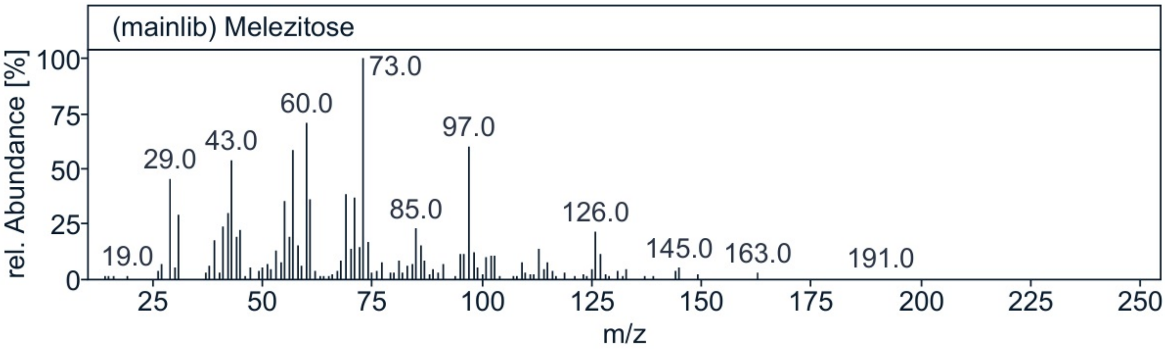

Supplementary Figure S2

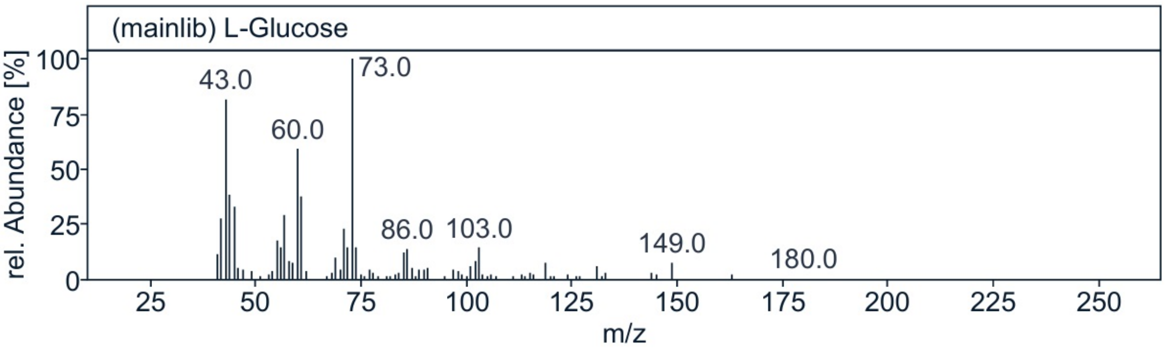

Supplementary Figure S3

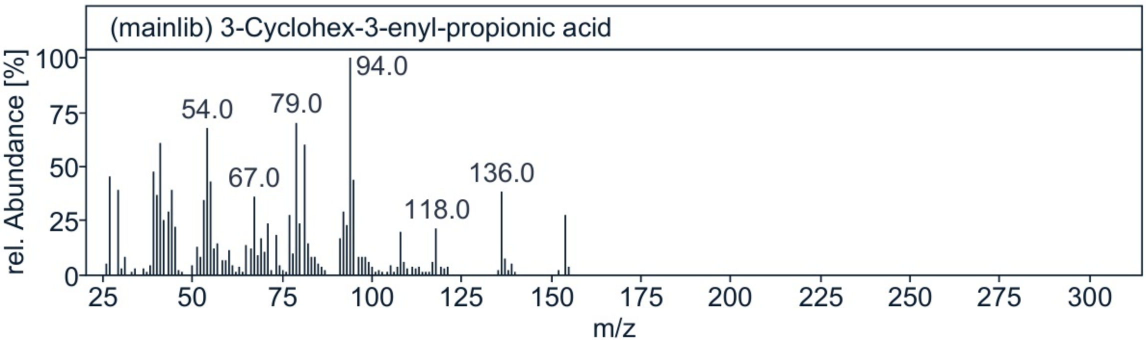

Supplementary Figure S4

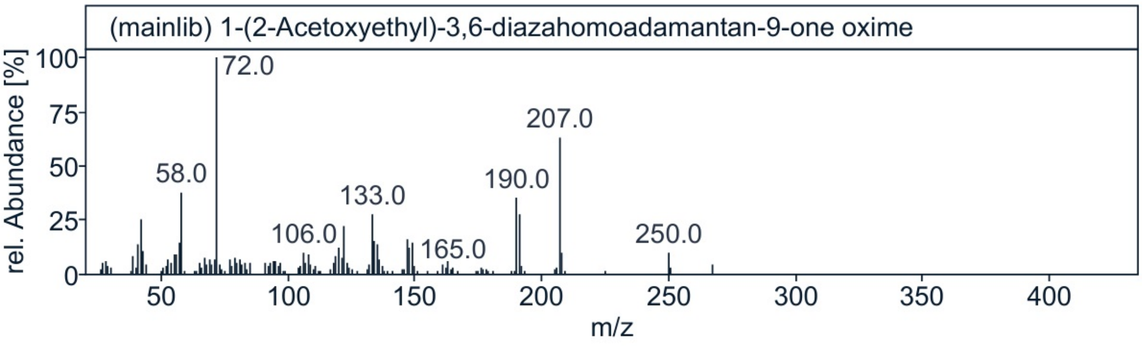

Supplementary Figure S5

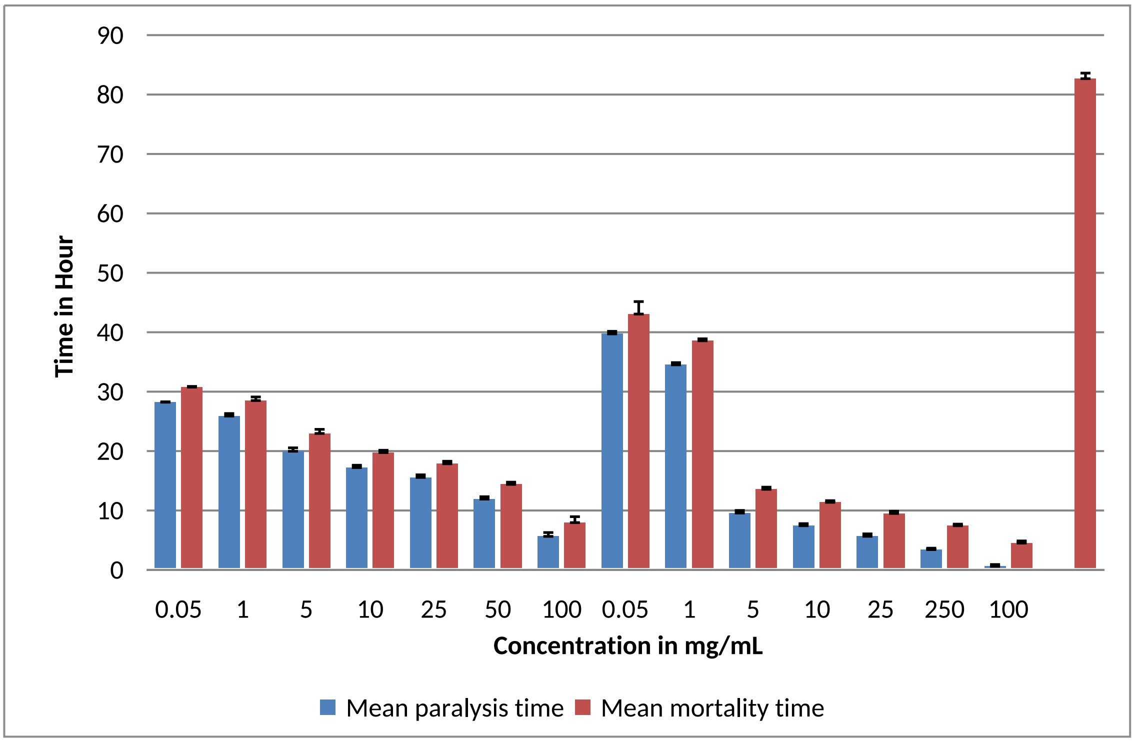

Supplement: Supplementary file 1 [file mmc1.pdf]
